# Matteo Carpano, Foundational Veterinary Scientist

**DOI:** 10.3201/eid3205.260029

**Published:** 2026-05

**Authors:** Donato Antonio Raele, Nicola Cavaliere

**Affiliations:** Istituto Zooprofilattico Sperimentale della Puglia e della Basilicata, Foggia, Italy

**Keywords:** bacteria, Aegyptianella pullorum, Carpano, piroplasm, birds, vector-borne infections, One Health, Italy

## Who is this scientist and what did he accomplish? 

Here is a clue: He first described *Aegyptianella pullorum,* a new genus of piroplasm infecting birds, in 1929.

A. Camillo Golgi

B. Giuseppe Sanarelli

C. Matteo Carpano

D. Adelchi Negri

E. Aldo Castellani 

Decide first, then see next page for the answer.

This is a photograph of Matteo Carpano (November 23, 1874–October 31, 1952) (Figure). Born in Manfredonia, Italy, Matteo Carpano emerged as one of Italy’s most influential veterinary scientists, bridging early 20th Century microbiology with principles that today align closely with the One Health paradigm, a holistic approach that recognizes the interdependence of human, animal, and environmental health. 

**Figure:**
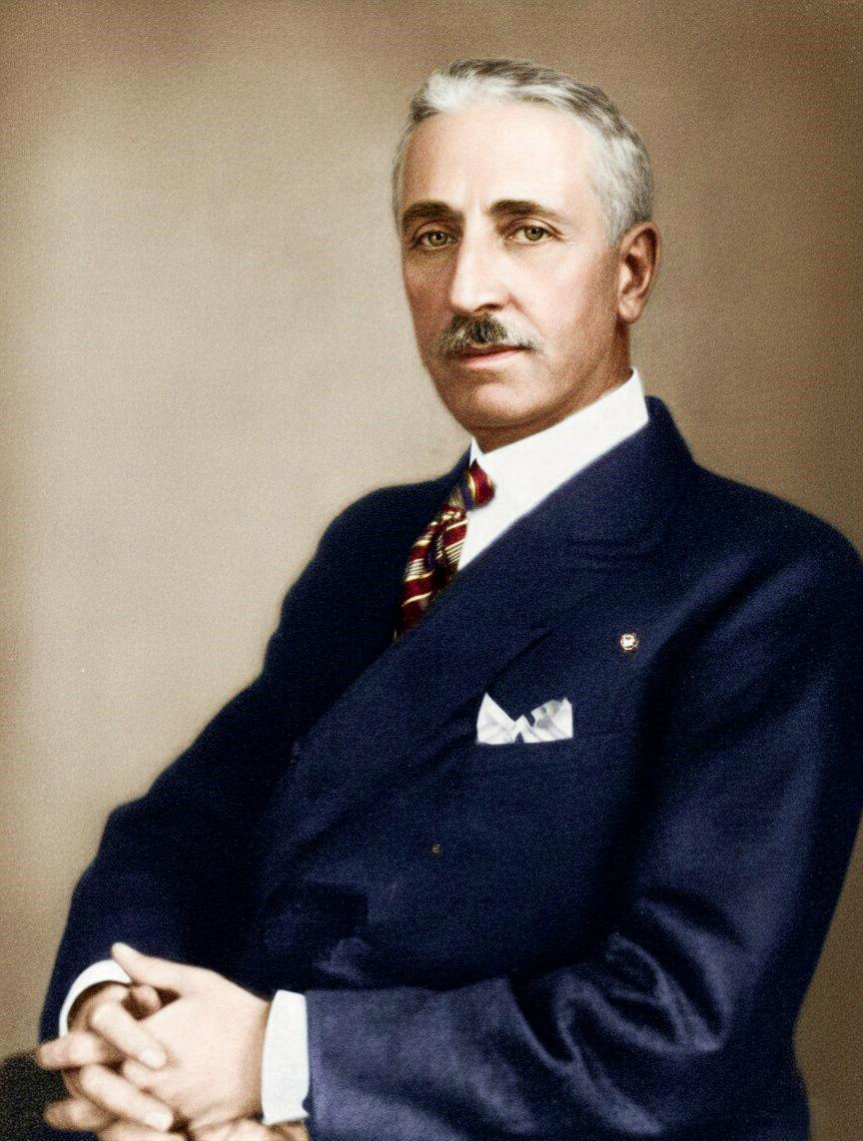
Matteo Carpano, 1930. Source: Historical archives of the city of Manfredonia, Italy.

After graduating with top honors in veterinary medicine from the University of Naples, where he received the prestigious Gasparini Prize, Carpano ranked first in the national competition for permanent military veterinary officers. His military career, which culminated in the highest rank of the Corps, was defined not by administrative duties but by an unwavering dedication to laboratory research, teaching, and scientific innovation.

During 1911–1928, Carpano served as director of the Military Veterinary Bacteriology Laboratory in Rome, transforming it into one of the most advanced centers of veterinary microbiology in Europe. His research spanned bacteriology, parasitology, tropical pathology, and emerging infectious diseases, fields in which he consistently demonstrated both scientific rigor and remarkable intuition. The main hall of the laboratory now bears his name as a testament to his enduring impact.

Carpano’s early fieldwork took him far beyond Italy. In 1903, he helped establish the Serum-Vaccinogen Institute in Eritrea to combat rinderpest virus. Despite contracting malaria and amoebic dysentery, he continued his investigations, even observing pathogen evolution on his own body. That extreme example of scientific dedication earned him the admiration of Governor Ferdinando Martini. Later, in Cyrenaica, Libya, he identified a bovine infection as a form of coast fever, correctly attributing it to *Theileria annulata*; that discovery contributed to improved diagnostic and control strategies in North Africa (*1*).

His most productive international period unfolded in Egypt during 1928–1938, where he served as bacteriologist and chief pathologist for the government of Egypt after winning an international competition. In Cairo, he described anthrax infection in birds (*2*,*3*), identified a new *Corynebacterium* species affecting camels (*4*), and documented new species of ciliated protozoa in horses (*Bertolinella intestinalis*), expanding scientific understanding of pathogens in arid ecosystems (*5*). During those years, he also made the discovery that would forever link his name to parasitology: *Aegyptianella pullorum*, a new genus of piroplasm infecting birds. That finding remains one of the cornerstones of early protozoan taxonomy (*6*). 

His contribution to entomology and vectorborne diseases was also substantial, especially in the study of leishmaniasis (*7*). and piroplasmosis (*8*,*9*). Although phlebotomine sand flies (*Sergentomyia antennata*) was originally described in 1912 (*10*), Carpano’s 1930 work remains fundamental to the scientific literature because it provided the first detailed morphologic descriptions and illustrations of *S. antennata* sand fly populations in Egypt, which helped to establish local variants and their ecology (*7*). Italy’s presence in Eritrea and later in Egypt reflected broader national ambitions for prestige and influence; its professionals were often showcased as embodiments of Italian scientific expertise abroad. Within that context, Carpano was presented internationally as a distinguished technical authority, yet his work remained strictly nonpolitical and was consistently well received by both local communities and governmental authorities.

His career was not without hardship. During his early years in Eritrea, Carpano suffered a devastating personal loss. His wife, Angelina, died in Asmara during an epidemic of typhus, at a time when he himself was deeply engaged in studying infectious diseases affecting both humans and animals in the African colonies. Professional tragedy struck decades later; much of Carpano’s extensive archive of work in Africa and the Mediterranean region was destroyed during the 1941 British occupation of Eritrea and subsequent institutional neglect in Italy. Shifting veterinary research priorities led to the loss of his pioneering protozoologic work, which was unjustly dismissed as obsolete. Yet his scientific output remained extraordinary; reports were translated into multiple languages and featured in leading international journals. He contributed extensively to the *Journal of Parasitology* and to the *Treccani Italian Encyclopedia*, further cementing his role as a foundational figure in veterinary science.

Carpano’s influence extended far beyond his own discoveries. Working across rural and periurban regions of southern Italy, he was among the first to recognize that zoonotic infections were shaped by environmental pressures, livestock management, and the movement of companion animals. His investigations into vectorborne pathogens revealed ecologic interactions between arthropod vectors and Mediterranean animal populations that had previously gone unnoticed. Although he did not use the term, his work anticipated the modern One Health framework, demonstrating how animal, human, and environmental health are inseparably linked. Those insights profoundly shaped veterinary public health in Italy and Europe. Carpano advocated for coordinated surveillance systems, improved diagnostic laboratories, and interdisciplinary training programs. As an adviser to the Ministry of Health, he helped develop national guidelines for vectorborne disease monitoring and strengthened collaborations between veterinary institutes and public health agencies. Many of those structures, especially the Istituti Zooprofilattici Sperimentali, still operate according to principles he championed.

His commitment to education was equally transformative. Carpano founded regional training centers for veterinarians in Puglia and Basilicata regions and mentored a generation of scientists who would become leaders in epidemiology, entomology, and infectious disease research. His emphasis on field observation, ecologic reasoning, and cross-species disease modeling encouraged young researchers to view zoonoses not as isolated outbreaks but as dynamic processes shaped by climate, human behavior, and animal health. Several of his students later credited him as the defining influence of their scientific careers.

Carpano’s legacy continued to evolve long after his death in Rome. He acknowledged critiques that early surveillance programs focused too heavily on livestock while underestimating wildlife ecology or socioeconomic vulnerability. For him, veterinary public health was a discipline that had to evolve continuously, integrating new knowledge and ethics considerations.

Today, research inspired by Carpano continues to advance understanding of vectorborne diseases in Mediterranean environments, including canine leishmaniasis, tickborne rickettsioses, and emerging arboviruses. His work has contributed to improved diagnostic tools, risk-mapping systems, and community-based prevention strategies, strengthening Italy’s capacity to respond to zoonotic threats.

Matteo Carpano’s life illustrates how veterinary science can shape public health, scientific discovery, and community resilience. His pioneering work, from early bacteriological studies in Africa to modern One Health approaches, demonstrates that understanding zoonotic diseases requires an integrated vision of animals, humans, and the environment. As global health challenges grow increasingly complex, future generations might well regard Carpano not only as a distinguished Italian veterinarian but also as one of the architects of modern interdisciplinary disease prevention.

To honor his memory, the city of Manfredonia named a street after him and erected a bronze bust in the Villa Comunale, sculpted by Luigi Schingo. Yet his most enduring monument remains the scientific legacy that continues to guide veterinary and public health research today.
